# Severe Cognitive Dysfunction and Occupational Extremely Low Frequency Magnetic Field Exposure among Elderly Mexican Americans

**DOI:** 10.9734/BJMMR/2014/7317

**Published:** 2014-04-16

**Authors:** Zoreh Davanipour, Chiu-Chen Tseng, Pey-Jiuan Lee, Kyriakos S. Markides, Eugene Sobel

**Affiliations:** 1 Department of Neurology, Keck School of Medicine, University of Southern California, Los Angeles, CA 90033, USA; 2 Department of Preventive Medicine, Keck School of Medicine, University of Southern California, Los Angeles, CA 90033, USA; 3 Department of Preventive Medicine and Community Health, University of Texas Medical Branch, Galveston, Texas 77555-1153, USA

**Keywords:** Severe Cognitive Dysfunction, Dementia, Occupational Exposure, Extremely Low Frequency Magnetic Fields, Elderly Mexican Americans, Hispanic Established Population for the Epidemiologic Study of the Elderly (H-EPESE), Mini-Mental State Exam

## Abstract

**Aims:**

This report is the first study of the possible relationship between extremely low frequency (50–60 Hz, ELF) magnetic field (MF) exposure and severe cognitive dysfunction. Earlier studies investigated the relationships between MF occupational exposure and Alzheimer’s disease (AD) or dementia. These studies had mixed results, depending upon whether the diagnosis of AD or dementia was performed by experts and upon the methodology used to classify MF exposure.

**Study Design:**

Population-based case-control.

**Place and Duration of Study:**

Neurology and Preventive Medicine, Keck School of Medicine, University of Southern California, 2 years.

**Methodology:**

The study population consisted of 3050 Mexican Americans, aged 65+, enrolled in Phase 1 of the Hispanic Established Population for the Epidemiologic Study of the Elderly (H-EPESE) study. Mini-Mental State Exam (MMSE) results, primary occupational history, and other data were collected. Severe cognitive dysfunction was defined as an MMSE score below 10. The MF exposure methodology developed and used in earlier studies was used.

**Results:**

Univariate odds ratios (OR) were 3.4 (*P*< .03; 95% CI: 1.3–8.9) for high and 1.7 (P=.27; 95% CI: 0.7–4.1) for medium or high (M/H) MF occupations. In multivariate main effects models, the results were similar. When interaction terms were allowed in the models, the interactions between M/H or high occupational MF exposure and smoking history or age group were statistically significant, depending upon whether two (65–74, 75+) or three (65–74, 75–84, 85+) age groups were considered, respectively. When the analyses were limited to subjects aged 75+, the interactions between M/H or high MF occupations and a positive smoking history were statistically significant.

**Conclusion:**

The results of this study indicate that working in an occupation with high or M/H MF exposure may increase the risk of severe cognitive dysfunction. Smoking and older age may increase the deleterious effect of MF exposure.

## 1. INTRODUCTION

Cognitive dysfunction and subsequent dementia is a common health-related problem among the elderly. For example, there are over 4 million Americans with Alzheimer’s disease (AD), the most common of the dementias; the chance of eventually developing AD once a subject reaches age 65 is over 10%, but varies depending upon apolipoprotein E (ApoE) allele status and other susceptibility factors [e.g. 1–5].

There are now twenty-one published studies of extremely low frequency electromagnetic field (ELF MF or simply MF) exposure and AD or dementia [6–26]. Twelve of these studies can be considered somewhat positive and nine can be considered negative. We note that four of the negative studies have used ELF MF exposure classifications that often results in subjects with rather high exposure being considered as having low exposure [12,17–19]. One of the negative studies used a cumulative ELF MF exposure classification, based only on job titles, which counts high ELF MF exposure when young the same as when older, which is not appropriate. Finally, one negative study used death certificate information cause of death (AD) and for occupation. Death certificate information is highly unreliable for both AD and occupation.

Seven of the negative studies and four of the positive or somewhat positive studies have been excluded from the review of individual studies below because the determinations of dementia and its subtyping, when attempted, were not performed by experts and/or because death certificates were used to determine dementia and its subtype. An additional negative study was excluded because only information concerning having worked in the aluminum industry or not was used as exposure information. The 4“positive” or somewhat positive studies are Feychting et al. [13], Håkansson et al. [14], Park et al. [15] and Johansen [16] and the 8 “negative” studies are Savitz et al. [17,18], Noonan et al. [19], Salib and Hillier [22], Schulte et al. [23], Seidler et al. [24], Sorahan and Kheifets [25] and Stampfer [26].

The other nine published studies have both diagnoses and MF exposure performed by experts [6–12, 20–21]. Eight have reported at least some association between AD and having a “primary” or last occupation with significant MF exposure [6–11, 20–21]. In the ninth study, Graves et al. [12] reported finding no association between occupations they classified as likely to result in MF exposure and Alzheimer’s disease [12]. However, they used a very low cut-point for MF exposure, thus classifying numerous subjects as exposed who most probably had quite minimal occupational MF exposure.

In the four published studies of dementia and MF exposure in which there was expert diagnosis or at least the use of an established dementia battery [8,11, 20–21]. Three of these studies [8,11,21] found evidence for an association with significant (high) MF exposure. The fourth study [20] found an increased dementia risk among subjects below age75 at onset who experienced medium or high occupational MF exposure. We emphasize studies which used expert diagnoses or a dementia battery because an “informal” diagnosis of dementia, AD, or any other differential diagnosis will have a high false positive probability. This will bias the analyses of risk factors towards the null hypothesis. In fact, the published results using death certificate or non-expert diagnoses are inconsistent in their results [13–19].

There have been two recent meta-analyses published [27,28] related to AD and occupational ELF MF exposure [27–28].

- The earlier (2008) Garcia et al. study [27] ascertained published reports through sometime in April 2006. Fourteen studies were ascertained which utilized “standardized criteria for AD diagnosis”: 9 case-control; 5 cohort. The case-control and cohort studies were analyzed separately. The pooled odds ratio for the case-control studies was OR_pooled_ = 2.03, 95% CI = (1.38, 3.00). The pooled relative risk (RR) for the cohort studies was RR_pooled_ = 1.62, 95% CI = (1.16, 2.27). Statistical heterogeneity was judged to be moderate to high.
- The later Vergara et al. study [28] ascertained published reports through January 11, 2012. Using ELF MF imputed levels for each occupation the combined RR_random effects_ = 1.58, P = .012, 95% CI = (1.20, 2.08). The number of studies in this analysis was 15. The particular references were not specified. The number of cases used in this analysis was not specified.

The two Sobel et al. studies [6,7], the Davanipour et al. study [9], the Harmanci et al. study [10] and the current study used the same MF classification protocol, primarily developed by Bowman and colleagues [29] using individual work measurements for specific occupations. Other researchers have used different protocols. The original Bowman protocol appears to be more conservative than the others in that fewer occupations are considered to have medium or high MF exposure. While the classification protocol was based on MF measurements, there may be some other exposures, rather than MF, which are etiologically relevant and highly correlated with the occupational classification protocol used. However, no such exposure has been suggested. In addition, as presented in the Discussion section, there are two possible biologic mechanisms which provide a plausible explanation for the apparent relationship between long-term MF exposure and the development of Alzheimer’s disease.

The current study was undertaken to determine whether significant occupational MF exposure might be a risk factor for severe cognitive dysfunction among Mexican Americans. Other factors may be positively or negatively associated with dementia or cognitive dysfunction, e.g., smoking, alcohol consumption, stroke, education. These factors were also investigated. Baseline data from a population-based, longitudinal study are analyzed. Two of the positive studies [7,9] cited above included subjects with a dementia other than vascular dementia (VaD) as controls. The implications of the present findings for these studies are discussed in the Discussion section, below.

## 2. METHODOLOGY

### 2.1 Study Population

The study population consists of the subjects in the initial or baseline phase (Phase 1) of the Hispanic Established Population for Epidemiologic Studies of the Elderly (H-EPESE), a longitudinal study of a representative sample of Mexican Americans age 65 and older, which was conducted in the five southwestern states of Texas, New Mexico, Colorado, Arizona, and California during 1993 and 1994. The H-EPESE was modeled after the EPESEs conducted in New Haven, East Boston, rural Iowa and North Carolina [30]. An area probability sample design was developed by listing counties in these southwestern states by the number of Mexican American residents, in descending population order, so as to cover 90% of all Mexican Americans in that region. Moreover, counties not chosen through this method but which were at least 30% Mexican American were added to assure inclusion of small counties with a significant Mexican American population.

Census tracts and enumeration districts in the above counties were subsequently listed by the size of the Mexican American population. Three hundred census tracts were randomly selected as primary sampling units and provided clusters for door-to-door screening. Systematic procedures were used to list households for screening. Interviews were conducted with up to four Mexican Americans age 65 and older in each household. Eighty-five percent (85%) of the subjects contacted agreed to participate in the study.

In-home personal interviews were conducted by trained bilingual interviewers for 3,050 women and men. Of these 3,050 subjects, 2,873 were self-respondents and 177 (5.8%) needed a surrogate-respondent. A more complete description may be found in Markides et al. [31].

Appropriate IRB approvals were obtained.

### 2.2 Data

The data collected include sociodemographic characteristics, occupational information, education, smoking and alcohol consumption history (Hx), Hx of chronic medical conditions (e.g., stroke, heart attack) and lifestyle habits (e.g., smoking, drinking) and Mini-Mental State Exam score. Specific occupational informational items (obtained by self-response or from a knowledgeable person if necessary) were (i) “What kind of work have you done most of your life? (What was your job called?)”, (ii) “In what kind of business or industry did you work for most of your working life?”, (iii) If this job had a specific “job title”, the interviewer was to record the title; (iv) “Did you have a particular job title?” (the interviewer was to record the job title if the response was affirmative; (v) “What were your most important activities or duties (in the job you did for most of your working life)?”.

### 2.3 Definition and Assessment of MF Occupational Exposure

Data were obtained for the subject’s “occupation”, with no additional descriptors, e.g., primary, usual, last. The data collected included items relating to the type of work, the primary tasks and the subject’s job title. The criteria for occupational MF classification were the same as in Sobel et al. [6,7] and Davanipour et al. [9]: medium exposure was defined as average occupational MF exposure between 2 and 10 mG or intermittently above 10 mG; high exposure was defined as average occupational MF exposure above 10 mG or intermittently above 100 mG. All other occupations were classified as having low exposure. Occupations were classified (by ES) into low (L), medium (M) or high (H) MF-exposed occupations based on the occupational information provided, using published procedures [29]. Subjects with a lack of specifics about their occupation were classified as having a low MF occupation. ‘Homemaker’ and ‘housewife’ were classified as low MF occupations. Assignment was made blinded as to the Mini-Mental State Exam (MMSE) score. The occupations classified as likely resulting in medium or high MF exposure are provided in Table 1, along with the number of subjects with each occupation.

### 2.4 Cognitive Dysfunction - MMSE Score

A MMSE score below 10 was used to define cognitive dysfunction for this study. The MMSE is sensitive to ethnic group, education and other factors. Rather than use a conventional, adjusted cut-point (e.g., Mungas et al. [32]), a score sufficiently low to essentially insure cognitive dysfunction was used. Subjects who could not complete the MMSE due to a hearing, visual or physical problem were excluded from the analyses unless their partial score was low enough to insure a total score below 10, assuming that they would have responded to all unanswered questions correctly.

### 2.5 Education

For descriptive purposes, educational attainment was classified as follows: no formal schooling through grade 5; completion of any grade from 6 through 9; 10 or more years of schooling. However, for the odds ratio analyses, education was dichotomized as below a 12th grade education versus having at least completed high school.

### 2.6 Smoking and Alcohol Consumption

Smoking and alcohol consumption histories were also assessed. Data on smoking and alcohol consumption are comparable to data from the Hispanic HANES [33,34]. Subjects were classified into ever having smoked regularly versus non-smokers, i.e., everyone else. Consumption of alcohol data was simply obtained as “ever” versus “never”.

### 2.7 Chronic Illnesses

Histories of heart attack and stroke were queried. Subjects were asked if they had ever been told by a doctor that they had had a stroke or heart attack.

### 2.8 Statistical Analyses

Logistic regressions to estimate odds ratios (OR) were performed using SAS. The two categories of each dichotomous variable were assigned numerical values of 0 (no) and 1 (yes). The categories for the ordinal variables were assigned consecutive integer values beginning with 0 for the reference category (e.g., age group). For multivariate logistic regression, stepwise, forward inclusion of variables was used to build a model, with a P-value of 0.05 used to enter or remain in the model. In addition to MF exposure, gender, age, education, family income and histories of heart attack, stroke, alcohol use and ever having regularly smoked cigarettes, along with all two-way interactions, were considered for the multivariate regressions. Thus, an explanatory variable, e.g., high MF exposure was considered to be statistically significance if the associated P-value of the variable is less than or equal to (≤) 0.05. Analyses were carried out for low vs medium/high and low vs high MF exposure classifications, with both 2 and 3 age group classifications and for those aged 75+. The age group classifications were (i) 65–74, 75–84, 85+, and (ii) 65–74, 75+. The 75–84 and 85+ age groups were combined because of the small number of MF exposed subjects among those 85+.

## 3. RESULTS AND DISCUSSION

### 3.1 Descriptive Statistics

Table 2 (next page) provides the distributions among the study subjects by gender, age, education, family income, ever having smoked regularly, ever having consumed alcohol, and histories of myocardial infarction (MI) and stroke. Except for family income, the percentage of subjects for which the requested information is missing is quite small. 57.7% of the subjects were women. 65.6% were 65–74 years old, 27.3% were 75–84 and 7.0% were 85+. We therefore have combined the 75–84 and 85+ age groups in some analyses. 3015 (98.9%) of the 3050 subjects had occupational information. All were classified with respect to MF exposure. The distribution of occupational MF exposure was as follows: low − 91.8%; medium − 4.7%; high − 3.5%. Among women, 76.7% of the medium or high exposed occupations were classified as high. The corresponding percentage for men was only 14.1%. This rather significant difference is due to the relatively large number of seamstresses and few other occupations with “large” numbers of women or men which are recognized as resulting in medium or high MF exposure (Table 3). 2873 (94.2%) of the subjects had a MMSE exam which could be scored as being below 10 or not. 1.7% of these subjects had a MMSE score below 10. The overall educational level of the study population was low: 61.7% had 5 or fewer years of schooling, 25.1% had 6–9 years of schooling and 13.2% had at least attended senior high school. 2844 (93.2%) subjects had both occupational information and an MMSE score. For these subjects, the distributions of age, gender, education, family income, histories of heart attack and stroke, smoking and alcohol consumption histories, MMSE score, and MF classification were, not surprisingly, very similar to the distributions for the entire sample (Table 2).

### 3.2 Univariate Odds Ratios – MF Exposure

MF occupational exposure classification and MMSE statistics by age group and gender are provided in Table 3 (below), while univariate odds ratios are given in Table 4. The rates of MMSE scores below 10 were 1.5% for those with a low MF exposure occupation, 0.7% for those with a medium MF exposure occupation and 5.0% for those with a high exposure occupation. The unadjusted odds ratios were 1.7 (P=.27; 95% confidence interval (CI): 0.7–4.1) for low vs medium/high (M/H) MF exposure and 3.4 (P<.03; 95% CI: 1.3–8.9) for low vs high MF exposure. Among the other exposure variables considered, only age, income and a history of stroke were significantly related to a low MMSE score.

### 3.3 Multivariate Model Odds Ratios: Main Effects

Age, gender, income, education, smoking and alcohol consumption history, and stroke and heart attack history were included in the stepwise, forward inclusion multivariate analyses. Table 5 (next page) provides the results of the main effects, using logistic regression analyses for low vs medium-to-high MF occupations and for low vs high MF occupational exposure, separately. When subjects aged 65+ were classified into 2 or 3 age groups, the results were quite similar. With low vs M/H MF among the exposures in the stepwise inclusion analysis, only age group and a history of stroke were significantly related to a low MMSE score. However, when low vs high MF exposure was a candidate variable, MF exposure was also significant with ORs of 3.7 and 3.3, depending upon whether 3 or 2 age groups were used. Because very few MF-exposed subjects were in the 65–74 age group (and none had a low MMSE score), analyses with only those subjects aged 75+ were also conducted. In these analyses, stroke and smoking histories were significant risk factors for all subjects. However, when the analyses were limited to those with either a high or low MF-exposed occupation, MF exposure was also a significant risk factor, with an OR of 5.8. It should be noted that the ORs for age group, stroke history and smoking history did not change much when analyses were limited to those with either low or high MF-exposed occupations, that is, when considering the risk associated with a high MF-exposed occupations rather than a high or medium exposed occupation.

### 3.4 Multivariate Model Odds Ratios: Main Effects and Interactions

Table 6 (below) provides the results for the main effects and interactions models. For these analyses, the significant risk factors were primarily interaction terms, specifically MF × age group, MF × smoking history, stroke history × age group and smoking history × age group. Neither MF exposure nor smoking history entered into the model as a main effect. When three age groups were used in the analyses and when the analyses were limited to those aged 75+, the interaction between MF exposure and smoking history was statistically significant. This was true for analyses using M/H MF exposure and for analyses using only high MF exposure. When two age groups were used in the analyses, the interactions MF × age group and smoking history × age group were significant. The somewhat different results may be due to the relatively smaller sample in the 85+ age group. The MF × smoking history interaction implies greater risk associated with MF exposure among current or ex-smokers, which may have biological plausibility (See Discussion section, 3.6.).

### 3.5 MMSE Cut-Point and Odds Ratio Stability

The cut-point of 10 was chosen both *a priori* and because it was low enough to essentially insure severe cognitive dysfunction among those scoring below the cut-point. However, we repeated these analyses for successively larger cut-points to consider the stability of our results. The significant explanatory variables and ORs were stable through a cut-point of 15 (data not shown).

### 3.6 DISCUSSION

#### 3.6.1 General considerations

The Hispanic EPESE is a large structured random sample of Mexican Americans aged 65+ living in California, Texas, Arizona, New Mexico and Colorado. It is representative of approximately 90% of the Mexican Americans in this age group living in these states. The MMSE is an accepted screening instrument for dementia within numerous ethnic groups, with the cut-point for further diagnostic evaluation depending upon age, education and ethnicity. For this study, we chose 10 as the cut-point (<10 vs ≥10), prior to looking at the distribution of MMSE scores, because a score below 10 has a very low false positive probability of identifying severe cognitive dysfunction. Some demented subjects are, of course, likely to be included among those with scores of 10 or above, thereby somewhat biasing the expected value of the OR estimator towards unity.

There were only 35 (1.1%) subjects who did not have any recorded information concerning occupation. 177 (5.8%) subjects did not complete the MMSE. Only 206 (6.8%) were missing occupational information or an MMSE score.

The underlying cause of the dementia among those with a MMSE score below 10 is not available. In general, among subjects whose dementia is not caused by a major stroke(s), Alzheimer’s disease accounts for about two-thirds of differentially diagnosed dementia, with VaD accounting for perhaps 20%. In the H-EPESE population, 10 (6.1%) of the 165 subjects with a stroke history, a MMSE score and occupational information, had a MMSE score below 10.

#### 3.6.2 Biological plausibility

##### MF and AD

A majority of dementia subjects have Alzheimer’s disease. Based on studies in which the diagnosis of AD was made by experts, working in an occupation with likely MF exposure after, say, age 45 may be etiologically related to development of AD [6–11]. Sobel and Davanipour [35] presented a biologically plausible hypothesis relating MF exposure to AD. Specifically, long-term significant MF exposure may cause increased production of amyloid beta (Aβ) in the brain and/or peripherally, with subsequent transportation of peripheral Aβ across the blood brain barrier. This increase in Aβ within the brain may be sufficient to overwhelm the brain’s ability to breakdown Aβ and/or transport the Aβ out of the brain, leading to the development of AD. This hypothesis has not yet been directly tested. However, in a longitudinal cohort study, Mayeux et al. [36] found that higher serum levels of Aβ_1-42_ were prognostic of development of AD among a sample of cognitively normal elderly subjects at baseline [36]. In addition, Noonan et al. have published data suggesting that MF exposure may up-regulate serum Aβ levels in a dose-dependent manner [37].

There is evidence for the plausibility of a second biological hypothesis. Research has indicated that (1) melatonin *in vitro* inhibits the neurotoxicity of Aβ [38–41] and (2) long-term exposure to moderate or high levels of occupational or residential MF exposure can cause down regulation of melatonin production [42–49]. Thus, chronically low levels of melatonin production may be etiologically related to AD incidence. Melatonin also is an important scavenger of reactive oxygen and nitrogen species [50]. Thus, dementias which are associated with neuronal death, perhaps due to oxidative stress, may, in part, be caused by chronic low levels of melatonin.

##### MF and VaD

A biologic process relating MF exposure to VaD is obscure. However, a paper by Savitz et al. [51] indicates that there may be a relationship between death due to MI and MF exposure among electric utility workers. A high proportion of the MIs were due to ventricular fibrillation (A Sastre, personal communication, 1999). Perhaps MF exposure may also induce atrial fibrillation leading to emboli causing small infarcts in the brain - a likely risk factor for VaD - particularly among smokers. For example, as noted, long-term MF exposure has been associated with decreased melatonin production and melatonin has shown capabilities of increasing cardiac electrical stability, lowering vulnerability to fibrillation, and protecting against ischemic injuries [52–54]. A more recent study by Sahl et al. [55], however, did not find any relationship between MI and MF exposure.

##### Smoking and AD or Dementia

A relationship, if any, between smoking and AD or dementia incidence has not yet been established. Some studies indicate that smoking may be a risk factor, some studies indicate that it may be protective, and other studies found it to be of no significance [56–61]. For example, the Wang et al. [57] study found a possible protective effect using cross-sectional data and a possible deleterious effect using longitudinal data. The latter finding was “replicated” in the longitudinal Rotterdam Study (Reitz et al. [61]). Reitz et al. found (i) a statistically significant increased risk of dementia and AD among smokers at baseline after a mean of 7.1 years of follow-up, (ii) that the increased risk was “restricted” to subjects without the ApoE ε4 allele and (iii) there was no increased risk among subjects who smoked but had stopped prior to baseline. With respect to the “restriction” in (ii), among subjects without an ε4 allele, the hazard ratios for incident dementia and incident AD were significant at the 0.05 level, while among subjects with at least one ε4 allele, the hazard ratios were very much non-significant.

For the comparison between low and medium or high MF exposed occupations among those aged 75+, we found that the interaction between MF exposure and smoking was a significant risk factor for dementia. Neither main effect entered into the model. It is thus possible that differences in previous study results for smoking are caused by differences in the occupational makeup of the study populations.

#### 3.6.3 Occupational pesticide exposure (Farming/Forestry)

There is some evidence that significant pesticide exposure, primarily from farming, is associated with decreased cognitive functioning AD. Pesticide exposure is considered common only in farming and perhaps forestry. Among the occupations with medium or high MF exposure (Table 1), only ‘machine operator’ (n=27) and ‘wood cutter; machinery repair - forestry’ (n=1) could possibly include working on a farm or in forestry. ‘Machine operator’ might represent farm and forestry workers who frequently drove heavy equipment or used power saws and other power equipment which could conceivably expose them to medium levels of MF. However, in this study, all subjects whose primary occupation was recorded as ‘farm worker’ or ‘forestry worker’ were classified as having low MF exposure. Consequently, only a very small proportion of the 141 subjects with medium MF exposure could possibly be presumed to have been exposed to pesticides. None of high MF exposed occupations could be reasonably expected to have also resulted in important levels of pesticide exposure. Therefore, the OR estimates related to either M/H MF or H MF exposure could not have been importantly affected by significant levels of pesticide exposure.

In addition, pesticide exposure has not been shown to be strongly related to cognitive function impairment. Four well designed, recent, and “positive” studies of cognitive function and pesticide exposure are reviewed below. In summary, the results of these studies are as follows:

- Tyas et al. [62] – AD RR = 1.45 (P>0.05);
- Baldi et al. [63] – AD adjusted RR (women) = 0.89 (P>0.05), AD adjusted RR (men) = 2.39 (P<0.05);
- Norlaily et al. [64] – dementia OR = 5.9 (P<0.01);
- Steenland et al. [65] – mean decrease in MMSE = 1.35 points (P=0.01).

Details of these studies are provided below:

- Tyas et al. [62] used the longitudinal Manitoba Study of Health and Aging (MSHA) to evaluate possible risk factors for probable/possible AD. The study included the 694 subjects who were cognitively intact at baseline (1991/1992, based on the modified MMSE) and who (a) completed a risk factor questionnaire at baseline, (b) completed the modified MMSE exam at follow-up (1996–1997), (c) did not have cognitive impairment due to something other than AD and (d) whose AD status (yes/no) was known. There were 36 (5.2%) probable/possible AD cases among the 694 subjects. Eight occupational exposure factors were considered: pesticides/fertilizers, defoliants/fumigants, inks/dyes, paints/stains/varnishes/gasoline/fuels/oils, solvents (degreasers), liquid plastics/rubbers/glues/adhesives. Exposure was dichotomous: ever vs never. Logistic regression, with adjustment for age, gender, and education, used to estimate the relative risk. Only defoliants/fumigants had statistically significant RR: 4.35, 95% CI=(1.05,17.90). It should be noted that exposure information was only available for between 26 and 28 of the AD cases, depending upon the exposure category. It should also be noted that about 25% of the study subjects were farmers and the RR for “ever having been a farmer” was statistically significant. However, when defoliant/fumigant exposure (yes/no) was included in the analysis model, having been a farmer was no longer significant.
- Baldi et al. [63] studied pesticide exposure and neurodegenerative diseases among 1,507 elderly French subjects, using the PAQUID Study. Occupational exposure data were collected only at the 5-year follow-up. Pesticide exposure was classified as “null” or “non-null” based on job titles. Nineteen job titles were considered to be associated with “non-null” pesticide exposure. Three hundred twenty (21.2%) of the 1507 subjects were considered to have had exposure. Cumulative exposure was estimated for 228 (71.3%) of the 320 exposed subjects. The median exposure duration was 28 years, while the median time since the end of exposure was 20 years. Analyses were performed for men, women and both genders and were adjusted for smoking and education. Eighty-seven (86.1%) of the 101 farmers and 101 (31.6%) of the blue-collar workers were considered to have had pesticide exposure. For both men and women, adjusted RRs were non-significant (P>0.05) for “main job in agriculture”, “rural residency”, “residency in a district planted with vineyards”. For “occupational exposure”, the adjusted RR for women was non-significant. Men had a significant adjusted RR for “occupational exposure”: RR_adj_=2.39, 95% CI: (1.02, 5.63). The authors note that “previous studies failed to demonstrate an association” between pesticide exposure and AD.
- Norlaily et al. [64] studied dementia in elderly patients in an out-patient clinic in Malaysia using a cross-sectional design. Among the out-patients at the hospital clinics, subjects who were 65 or older and did not have a severe mental disorder, mental retardation, were neither deaf, dumb or blind and who had not already been diagnosed as having dementia were eligible. All 399 eligible subjects agreed to participate. The validated Malay versions of the MMSE and the Elderly Cognitive Assessment Questionnaire (ECAQ) were administered to the literate subjects and illiterate subjects, respectively. Subjects and caregivers provided potential risk factor exposure information. Literate subjects with an MMSE score 17 or below and illiterate subjects with a ECAQ score of 5 or below were considered to have cognitive impairment and were further evaluated based on the Diagnostic and Statistical Manual IV (DSM-IV; American Psychiatric Association, 1994) criteria. Forty-seven of the 399 original subjects were considered cognitively impaired. Thirty-nine (83%) of these 47 subjects participated in the full clinical (Phase 2) component of the study. Ten (2.6%) of the 391 evaluable subjects were considered demented. Fischer’s exact test was performed for pesticide exposure (yes/no). The estimated OR was 5.9, P<0.01.
- Steenland et al. [65] studied occupational pesticide exposure and screening tests for neurodegenerative diseases in 400 elderly Cost Ricans who attended a “routine annual free medical exam. (Two percent (2%) of the subjects were age 60–64, the remainder were 65+.) The area is currently partly agricultural and partly on the outskirts of San Jose. Three hundred fifty-three (88%) of the 400 subjects were administered the MMSE and the United Parkinson’s Disease Rating Scale (UPDRS). Of the 163 subjects who “failed” either test, 144 (88%) were examined by a neurologist, who made a diagnosis of Parkinson’s disease, AD, Mild Cognitive Impairment (MCI)or essentially “other” (which included “mixed dementia”), according to 2011 international criteria. Subjects also provided (evidently personally) pesticide information: ever worked in agriculture (yes/no); if yes, ever worked with pesticides (yes/no) and number of years worked with pesticides. Eighteen percent (18%) of the subjects reported occupational pesticide exposure. Two relevant analyses were conducted: a regression of MMSE (1) score on occupational pesticide exposure, age, gender, and education; (2) logistic regression with AD/MCI and occupational pesticide exposure as the dependent and independent variables, respectively. It should be noted that the AD subjects had to show up to the clinic to be in the study. The authors state that the expected number of AD subjects in their population was 21, with 14 attending the clinic. They could not provide any estimate for MCI subjects. They state that they have no reason to believe that AD subjects with pesticide exposure have a different likelihood of attending the clinic than AD subjects without such exposure.
  1. In the MMSE analysis, occupational pesticide exposure (yes/no) was associated with a decrease in the average MMSE score by 1.35 points, P=0.01, after adjustment for age, gender, and education. Without the adjustment, the difference was 1.0 points, P=0.01. For years of pesticide exposure, there was a statistically significant downward trend of 0.5 points per year of exposure. There was no significant effect on MMSE for agricultural work in general.
  2. Logistic regression, with AD/MCI as the dependent variable, did not demonstrate any excess risk related to occupational pesticide exposure, P=0.70 or any trends by years of pesticide exposure.

#### 3.6.4 Occupational psychosocial stress

There is a long history of research related to the effects of occupational stress factors, e.g., job demand, job control, job support, and job strain levels, on the subsequent development of cognitive impairment, dementia, AD and vascular dementia. Based upon the two studies [66,67] described below and other studies, job control and distress proneness (whether or not associated with a job) are important factors associated with psychosocial stress and subsequent development of dementia and AD. Low job control and high distress proneness, among other possible psychosocial factors are therefore possible risk factors the development of severe cognitive dysfunction. The association between job control/distress proneness and medium or high MF occupations versus low MF occupations, however have apparently not been studied.

Studies have found that chronic occupational M/H MF exposure can lead to increased amyloid beta production both in the brain and peripherally [68] and to lowered melatonin levels [69]. Increased amyloid beta and decreased melatonin increase the risk of AD and thus likely account for at least some of the risk of severe cognitive dysfunction. Note that peripheral amyloid beta can subsequently be transported to and through the blood brain barrier and thus enter the brain. It is also the case that chronic psychosocial stress may also lead to increased cognitive deficits in a rat AD model caused by amyloid beta dosing and that lowered melatonin levels appear to be associated with increased psychosocial stress. See, for example Rothman et al. [70], Alkadhi [71], Callaghan [72], Rimmele et al. [73] and Wirtz et al. [74]. Consequently, chronic occupational M/H MF and psychosocial stress may act jointly to increase the likelihood of severe cognitive dysfunction, dementia, and AD.

The two referenced studies [66,67] above are described below:

- In a longitudinal study, Wang et al. [66] found that compared to high job control, workers with moderate, limited and/or low job control were at increased risk of both dementia and AD. Adjusting for age, gender, and education, the hazard ratios were 1.8 or 1.9 (P<0.05) for dementia and 2.1–2.3 (P<0.05) for AD. For job demands, the differences between high and moderate, limited or low and between low and high-limited were not statistically significant. With respect to job strain, Wang et al. [66] found statistically significant differences (P<0.05) for active versus passive or high for both dementia and AD. Strata for job strain were defined as follows: active – high control/high psychosocial demands; low – high control, low psychosocial demands; passive – low control, low psychosocial demands and high – low control and high psychosocial demands. In this analysis, the hazard ratios were controlled for age, gender, education, depressive symptoms and vascular factors.
- Wilson et al. [67] studied chronic psychological distress and the risk of AD using the Rush Memory and Aging Project (RMAP). The RMAP is a longitudinal study designed to identify/evaluate clinical and pathologic factors related to various common chronic problems among the elderly. Study subjects were essentially volunteers from various communities in the Chicago area. Medical, social and work histories were obtained. Detailed annual examinations were conducted and, to the extent possible, brains were autopsied at death. Proneness to distress was evaluated using items from the Neuroticism Scale of the Neuroticism-Extroversion-Openness Inventory (NEO-I). Cognitive function was evaluated at annual visits using a detailed battery of common tests, excluding the MMSE. Progressive cognitive decline as a function of distress proneness was determined. Subsequently, the relationship of distress proneness to development of clinical diagnosable AD was evaluated by consideration of the level of cognition and the rate of cognitive decline at successive annual visits. Compared to subjects whose proneness to psychological distress was in the bottom 10^th^ percentile, subjects who were quite prone (top 10^th^ percentile) were (1) about 2.7 times more likely to develop dementia and (2) had a more rapid cognitive decline. Age, gender and education were unrelated to the association between distress proneness and subsequent AD. On the other hand, distress proneness was related to depressive symptomatology but not the number of symptoms. In addition, distress proneness was inversely related to cognitive, social and physical activity. Finally, distress proneness was not associated with specific AD brain pathologies after adjustment for age, gender and education, e.g., number of plaques and tangles or summary measures of tau-immunoreactive neurofibrillary tangles or amyloid-β-immunoreactive plaques. On the other hand, “proximate to death” distress proneness was statistically significantly related to (i) global cognition, (ii) episodic memory and (iii) perceptual speed but not to semantic, working memory or visuospatial ability.

#### 3.6.5 Inclusion of interaction terms without main effects in model development

In the main effects analyses (Table 4: individual main effect analyses and Table 5 joint main effect analysis), high MF exposure was statistically significant, but medium/high MF exposure was not significant. In the analysis using sequential or stepwise inclusion of main effects and interactions terms (Table 6), H MF and M/H MF interactions were statistically significant, but neither main effect was significant. Among the six models developed, the interaction term MF × Smoke Hx was significant for both M/H and H MF when 3 age groups or just 75+ were used and MF × Age Group was statistically significant when 2 age groups were used. Only Age Group (65–74, 75–84, 85+) and Stroke Hx were significant as main effects, twice for Age Group and twice for Stroke Hx.

Many researchers, including evidently some statisticians, argue that the main effects of each significant interaction term must be included in models. Others argue that the interaction terms should be considered independent of the associated main effects terms. In the present study, because variable inclusion was stepwise with P-values of 0.05 to enter or exit from the model, we know that when the interaction term is in the model and one or both of the main effect variables are not in the model that the excluded main effect variables had P-values to enter which were greater than 0.05. Thus, it is likely that their inclusion would have decreased or eliminated the “significance level” of the interaction terms.

It is important to note that, in the present study, both main effect only and main effect/interaction analyses have been conducted. The results of the main effect only models (Tables 4 and 5) and the model developed stepwise by allowing the inclusion of significant main effect and interaction effects (Table 6), demonstrate the following:

- high MF but not M/H MF, exposure is a statistically significant main effect when interaction terms are not considered;
- the interactions of either M/H MF or H MF with smoking history are statistically significant, but neither M/H MF nor H MF (as main effects) are significant when the analysis is restricted to subjects 75+ or to 3 age groups;
- the interactions of either M/H MF or H MF with age group are statistically significant, but neither M/H MF nor H MF (as main effects) are significant when only two age groups are considered;
- the interaction terms of either M/H MF or H MF with smoking history are statistically significant, but (again) neither M/H MF nor H MF (as main effects) are significant when the analysis is restricted to subjects aged 75+.

Thus, the analyses indicate that H MF and M/H MF occupational exposure is associated with severe cognitive function primarily among subjects who have a history of smoking and/or are 75+ at the baseline interview for the H-EPESE. Many earlier studies have demonstrated that both a history of smoking and older age are associated with a higher likelihood of decreased cognitive functioning, dementia, and AD [e.g., 75–78]. Consequently, the analytic results make sense. It should be noted that the P-values for H MF are quite a bit lower than the P-values for M/H MF in Tables 4–6.

## Figures and Tables

**Table 1:** Occupations classified as being likely to have resulted in medium or high MF Exposure

Medium MF Occupations – (N)	High MF Occupations – (N)
Beautician (2)	Cutter (clothing) (2)
Carpenter (34)	Power Plant Operator (1)
Clothes Inspector: Manufacturing Company (4)	Repair Sewing Machines (1)
Electric Lineman (1)	Seamstress (87)
Electrician (7)	Welder (14)
Electronics Technician (1)	
Electronic Assembler (2)	
Equipment Repair (7)	
Fabric Cutter (1)	
Foam Cutter (1)	
Forklift Operator (6)	
Furniture Maker (4)	
Machine Operator (27)	
Machinery Repair (3)	
Machinist (12)	
Newspaper Pressman (1)	
Presser: Clothing Manufacturing Company (2)	
Seamstress – Part-time (3)	
Sheet Metal Machine Operator (4)	
Shoemaker (5)	
Typist (2)	
Upholstery; Re-Upholstery (4)	
Welder – Part-time (5)	
Wood Cutter; Machinery Repair - Forestry (1)	
Wood Sander - Furniture (2)	

(N): Number

**Table 2:** Distribution of demographic, health, MMSE (outcome) and MF exposure variables

Variable	Category	All Subjects	Subjects with MMSE and MF Data

		Number (%)	Number (%)
Sex	Women	1761 (57.7)	1656 (58.2)
	Men	1289 (42.3)	1188 (41.8)
Age Group	65–74	2002 (65.6)	1898 (66.7)
	75–84	834 (27.3)	776 (27.3)
	85+	214 (7.0)	170 (6.0)
Education	0–5 Years	1853 (61.7)	1727 (61.5)
	6–9 Years	754 (25.1)	717 (25.5)
	10+ Years	395 (13.2)	364 (13.0)
	Missing	48 (---)	36 (---)
Family Income	< $5000	432 (16.3)	407 (16.2)
	$5,000 – $9,999	1129 (42.7)	1085 (43.3)
	$10,000 – $1,4999	649 (24.5)	605 (24.2)
	$15,000 – $19,999	299 (11.3)	276 (11.0)
	$20,000+	136 (5.1)	132 (5.3)
	Missing	405 (---)	339 (---)
Stroke	Yes	204 (6.7)	165 (5.8)
	No	2839 (93.3)	2674 (94.2)
	Missing	7 (---)	5 (---)
Heart Attack	Yes	333 (11.0)	298 (10.5)
	No	2703 (89.0)	2535 (89.5)
	Missing	14 (---)	9 (---)
Ever Smoked Regularly	Yes	1258 (41.4)	1179 (41.5)
	No	1783 (58.6)	1661 (58.5)
	Missing	9 (---)	4 (---)
Ever Consumed Alcohol	Yes	1393 (45.8)	1292 (45.5)
	No	1648 (54.2)	1548 (54.5)
	Missing	9 (---)	4 (---)
MMSE Score	< 10	49 (1.7)	45 (1.6)
	10+	2824 (98.3)	2799 (98.4)
	Missing	177 (---)	0 (---)
MF Index	Low	2769 (91.8)	2608 (91.7)
	Medium	141 (4.7)	135 (4.7)
	High	105 (3.5)	101 (3.6)
	Missing	35 (---)	0 (---)

**Table 3:** MMSE scores by age group and gender

Characteristic	MF Classification	MMSE Score (%)		
		< 10	≥ 10	Total
**Age Group**				
65–74	Low	12 (100.0)	1725 (91.5)	1737 (91.5)
	Medium	0 (0)	93 (4.9)	93 (4.9)
	High	0 (0)	68 (3.6)	68 (3.6)
75–84	Low	16 (76.2)	692 (91.6)	708 (91.2)
	Medium	1 (4.8)	36 (4.8)	37 (4.8)
	High	4 (19.0)	27 (3.6)	31 (4.0)
85+	Low	11 (91.7)	152 (96.2)	163 (95.9)
	Medium	0 (0)	5 (3.2)	5 (2.9)
	High	1 (8.3)	1 (0.6)	2 (1.2)
**Gender**				
Women	Low	25 (86.2)	1523 (93.6)	1548 (93.5)
	Medium	0 (0)	25 (1.5)	25(1.5)
	High	4 (13.8)	79 (4.9)	83 (5.0)
Men	Low	14 (87.5)	1046 (89.2)	1060 (89.2)
	Medium	1 (6.3)	109 (9.3)	110 (9.3)
	High	1 (6.3)	17 (1.5)	18 (1.5)

**Table 4:** Univariate risk factor analyses

Variable (Classes)	Odds Ratio	P-Value (exact)	95% CI (per Unit)
L vs H MF	3.4	< 0.03	1.3 – 8.9
L vs M/H MF	1.7	0.27	0.7 – 4.1
Gender: women = 1, men = 2	1.5	0.24	0.8 – 2.7
Age: 65–74 vs 75+	5.7	< 0.0001	2.9 –11.8
Education: < 12 vs ≥ 12	0.95	1.0	0.4 – 3.1
Income: < $10,000 vs ≥ $10,000	0.5	< 0.03	0.2 – 0.9
Heart Attack	1.7	0.27	0.7 – 3.7
Stroke	5.0	< 0.0001	2.3 –10.3
Ever Smoked Regularly	1.3	0.51	0.5 – 2.3
Ever Consumed Alcohol	0.8	0.64	0.4 – 1.5

**Table 5:** Stepwise logistic regression analysis of MF occupational classifications and MMSE score: main effects only

Variable (Classes)	Risk Factors in Model	Odds Ratio (per unit)	P-Value	95% CI
L vs M/H MF	Age Group	3.7	< 0.0001	2.2 – 5.0
65–74, 75–84, 85+	Stroke Hx	4.1	< 0.0002	2.0 – 8.7
L vs M/H MF	Age Group	5.0	< 0.0001	2.5 – 9.7
65–74, 75+	Stroke Hx	3.8	< 0.0004	1.8 – 8.0
L vs H MF	MF	3.7	< 0.02	1.3 – 10.1
65–74, 75–84, 85+	Stroke Hx	3.9	< 0.0004	1.8 – 8.4
	Age Group	3.4	< 0.0001	2.2 – 5.1
L vs H MF	MF	3.3	< 0.02	1.2 – 9.0
65–74, 75+	Stroke Hx	3.8	0.0005	2.4 – 9.4
	Age Group	4.8	< 0.0001	1.1 – 4.8
L vs M/H MF	Stroke Hx	4.9	< 0.0001	2.2 – 10.5
75+	Smoke Hx	2.4	< 0.03	1.1 – 4.9
L vs H MF	MF	5.8	0.002	2.0 – 17.4
75+	Stroke Hx	4.6	< 0.0002	2.0 – 10.4
	Smoke Hx	2.6	< 0.02	1.2 – 5.6

**Table 6:** Stepwise logistic regression analysis of MF occupational classifications and MMSE Score: main effects and interactions

Variable (Classes)	Risk Factors In Model	Odds Ratio (per unit)	P-Value	95% CI
L vs M/H MF	MF × Smoke Hx	3.2	< 0.02	1.2 – 6.8
65–74, 75–84, 85+	Age Group × Stroke Hx	3.0	< 0.02	1.7 – 5.4
	Age Group	3.0	< 0.0001	2.0 – 4.7
L vs M/H MF	MF × Age Group	3.0	< 0.02	1.2 – 7.9
65–74, 75+	Age Group × Stroke Hx	5.1	< 0.0001	2.3 – 11.4
	Age Group × Smoke Hx	3.7	< 0.0001	1.9 – 7.1
	Hearing × Gender (Women*)	2.5	< 0.02	1.2 – 5.2
L vs H MF	MF × Smoke Hx	8.9	< 0.0001	2.7 – 29.5
65–74, 75–84, 85+	Age Group × Stroke Hx	3.0	< 0.0001	1.6 – 5.4
	Age Group	3.0	< 0.0001	1.9 – 4.7
L vs H MF	MF × Age Group	6.8	0.0006	2.3 – 20.4
65–74, 75+	Age Group × Stroke Hx	5.0	< 0.0001	2.2 – 11.4
	Age Group × Smoke Hx	3.9	0.0002	2.0 – 7.4
	Hearing × Gender (Women*)	2.3	< 0.03	1.1 – 4.8
L vs M/H MF	MF × Smoke Hx	4.6	< 0.005	1.6 – 13.0
75+	Stroke Hx	5.0	< 0.0001	2.2 – 11.0
L vs H MF	MF × Smoke Hx	17.8	< 0.0001	4.6 – 69.1
75+	Stroke Hx	5.2	< 0.0001	2.3 – 11.8

* In the analyses, women were coded as 1 and men were coded as 0.

## ABBREVIATIONS

Aβ: amyloid beta
AD: Alzheimer’s disease
ApoE: apolipoprotein E
CI: confidence interval
DSM-IV: Diagnostic and Statistical Manual IV
ECAQ: Elderly Cognitive Assessment Questionnaire
ELF: extremely low frequency
H: high
H-EPESE: Hispanic Established Population for the Epidemiologic Study of the Elderly
Hx: history
L: low
M: medium
MCI: mild cognitive impairment
MF: magnetic field
M/H: medium or high
MI: myocardial infarction
MMSE: Mini-Mental State Exam
OR: odds ratio
RR: relative risk
UPDRS: United Parkinson’s Disease Rating Scale
VaD: vascular (multi-infarct) dementia
